# Effect of dietary tannin supplementation on cow milk quality in two different grazing seasons

**DOI:** 10.1038/s41598-021-99109-y

**Published:** 2021-10-04

**Authors:** R. Menci, A. Natalello, G. Luciano, A. Priolo, B. Valenti, G. Farina, M. Caccamo, V. Niderkorn, M. Coppa

**Affiliations:** 1 Department Di3A, University of Catania, via Valdisavoia 5, 95123 Catania, Italy; 2 Department DSA3, University of Perugia, Borgo XX Giugno 74, 06121 Perugia, Italy; 3 Consorzio per la Ricerca nel settore della Filiera Lattiero-Casearia e dell’agroalimentare (CoRFiLaC), Regione Siciliana, 97100 Ragusa, Italy; 4 INRAE, Université Clermont Auvergne, Vetagro Sup, UMRH, 63122 Saint-Genès-Champanelle, France; 5 Independent Researcher at INRAE, Université Clermont Auvergne, Vetagro Sup, UMRH, 63122 Saint-Genès-Champanelle, France

**Keywords:** Animal physiology, Fatty acids

## Abstract

Extensive farming systems are characterized by seasons with different diet quality along the year, as pasture availability is strictly depending on climatic conditions. A number of problems for cattle may occur in each season. Tannins are natural polyphenolic compounds that can be integrated in cows’ diet to overcome these seasonal problems, but little is known about their effect on milk quality according to the season. This study was designed to assess the effects of 150 g/head × day of tannin extract supplementation on proximate composition, urea, colour, cheesemaking aptitude, antioxidant capacity, and fatty acid (FA) profile of cow milk, measured during the wet season (WS) and the dry season (DS) of Mediterranean climate. In WS, dietary tannins had marginal effect on milk quality. Conversely, in DS, the milk from cows eating tannins showed 10% lower urea and slight improvement in antioxidant capacity, measured with FRAP and TEAC assays. Also, tannin extract supplementation in DS reduced branched-chain FA concentration, C18:1 *t*10 to C18:1 *t*11 ratio and rumenic to linoleic acid ratio. Tannins effect on rumen metabolism was enhanced in the season in which green herbage was not available, probably because of the low protein content, and high acid detergent fibre and lignin contents in diet. Thus, the integration of tannin in the diet should be adapted to the season. This could have practical implications for a more conscious use of tannin-rich extracts, and other tannin sources such as agro-industrial by-products and forages.

## Introduction

Extensive farming systems are characterized by a dietary imbalance along the year, as they are strictly depending on the climatic conditions^1^. In particular, the seasonal variations under Mediterranean climate cause the alternation of periods with different pasture availability, with implications on animal performance and product quality. For instance, dairy cows reared under traditional husbandry systems have higher milk yield, and protein and fat contents during the green season compared to the dry season^2^. In addition, grazing is reported to increase the contents of vitamins and aromatic compounds^3^, and the proportion of polyunsaturated fatty acids (PUFA) and conjugated linoleic acid^4^ in milk. On the other hand, young fresh herbage may cause an excess of degradable protein in the rumen with implications on protein metabolism efficiency and nitrogen excretion^5^.

To overcome these seasonal problems, farmers can adopt several strategies. For example, tannins are plant polyphenols used in ruminant farming as growth and health promoter. Many forages and agricultural by-products are naturally rich in tannins, especially in plant species characterizing marginal areas or dry habitats^6^, but tannins can be also added as dietary supplement for a better control of dose and quality. Thanks to their antimicrobial and protein binding activities, tannins are known to affect ruminal biohydrogenation (BH) and N metabolism, with potential positive consequences on milk quality and N emissions^7^.

However, the information available in literature does not clarify if and how the effects of dietary tannins might vary according to the season in extensive farming systems. In a recent study, a different response on in vitro rumen BH and fermentation was observed when tannin extracts were incubated with a green forage or a hay substrate^8^. Therefore, we hypothesized that a different effect of dietary tannins could be observed on cow milk quality when supplemented during the grazing season or the season in which diet is based on dry forages. Thus, the present study aimed to assess the effects of supplementing tannin extract to dairy cows grazing in two different seasons (spring and summer) under Mediterranean climate on the milk quality. We chose to fit into on-farm conditions to directly test the practical effects of dietary tannin extract.

The experimental design of studies focusing on dietary tannins generally provide for sampling at the end of the trial^9–12^. However, an earlier effect of dietary tannins could not be ruled out. Therefore, the present study was also designed to evaluate the effect of dietary tannins on milk quality from the beginning of the supplementation, throughout the experimental period, to have a deeper insight of the subject.

## Methods

### Experimental design, animals and diets

All the experiment was performed in accordance with relevant guidelines and regulations (following the ARRIVE guidelines^13^). All procedures were approved by the animal welfare committee (OPBA) of the University of Catania (UNCTCLE-0015295). The experimental design is detailed in a previous study^12^. Two experiments were performed in a commercial extensive farm located in an upland area of the Mediterranean island of Sicily, Italy (36° 57′ N, 14° 40′ E; altitude: 670 m). The first experiment was carried out during the wet season (WS), between March and April 2019, and the second one during the dry season (DS), in July 2019. In both experiments, 14 lactating dairy cows (Modicana breed) were divided into two groups (*n* = 7), namely control (CON) and tannin (TAN), balanced for average milk yield, and protein and fat contents recorded in the two days before the beginning of each trial, together with DIM, parity, and BCS, as reported in Menci et al.^12^. The groups were composed of different animals in WS and DS experiment. In WS, the cows were free to graze on 20 ha of spontaneous pasture. In DS, the cows were free to graze on 20 ha of dry stubble of an annual crop (vetch:oat:barley 40:40:20), and no fresh herbage was available. A detailed description of site, weather conditions, and pasture botanical composition is reported in Menci et al.^12^.

In both experiments, commercial pelleted concentrate was individually offered to cows in two equal meals just before milking, at a rate of 6.4 kg/head × day in WS and 9.6 kg/head × day in DS, following the farm routine. Pelleted concentrate was composed of: corn grain (420 g/kg), soybean meal CP 48% (250 g/kg), wheat middling (100 g/kg), corn flake (66 g/kg), carob germ (60 g/kg), carob pod (30 g/kg), beet pulp (30 g/kg), rumen protected fat (10 g/kg; Magnapac, Or Sell S.p.a.), Na_2_CO_3_ (10 g/kg), CaCO_3_ (10 g/kg), NaCl (8 g/kg), vitamins and minerals supplement (4 g/kg), urea (2 g/kg). Moreover, 2 kg/head of hay (vetch:oat:barley 40:40:20) was daily offered to cows in the same way as concentrate. The cows always completely consumed the offered concentrate and hay. The chemical composition of feedstuffs is shown in Table 1. In both WS and DS experiments, TAN cows daily received 150 g/head of a commercial tannin extract (Silvafeed ByProX; Silvateam), a mixture of chestnut (*Castanea sativa* Mill., 60%) and quebracho (*Schinopsis lorentzii* Engl., 40%) tannins, included in pelleted concentrate. Total phenolic compounds concentration in tannin extract was 688 g of tannic acid equivalents per kg of DM, with 90.2% of tannins, according to the method of Makkar et al.^14^. Basing on the potential intake capacity of experimental cows, estimated according to INRA method^15^, the tannin extract intake corresponded to 1% of estimated dry matter intake (DMI).

**Table 1:** Chemical composition of feeds used in wet season (WS) and dry season (DS) experiments.

Item	Concentrate	Hay	Pasture (only in WS)	Stubble (only in DS)
DM, g/kg	889	833	186	876
**Chemical composition, g/kg DM**				
CP	200	79	222	69
Ether extract	36	12	28	11
NDF	179	708	415	672
ADF	80	460	269	472
ADL	22	62	38	76
Ash	51	66	94	67
**Phenolic compounds, g TAeq**^**a**^**/kg DM**				
Phenols	5.2	5.2	14.2	5.4
Tannins	3.9	1.6	4.7	1.7
**Protein fractions**^**b**^**, g/100 g CP**				
A	15.1	37.3	37.1	22.3
B1	7.9	10.8	7.6	18.2
B2	59.6	12.2	21.4	20.3
B3	12.9	29.5	28.2	26.0
C	4.5	10.1	5.8	13.1
**Fatty acids, g/100 g fatty acids**				
C16:0	18.2	29.2	14.1	25.1
C18:0	9.2	6.0	2.4	6.2
C18:1 *c*9	16.8	9.4	3.0	6.5
C18:2 *c*9*c*12	36.2	27.0	12.1	21.1
C18:3 *c*9*c*12*c*15	1.8	16.0	52.2	21.6

^*a*^*TAeq* tannic acid equivalents.

^b^*A* NPN, *B1* buffer-soluble true protein, *B2* neutral detergent soluble protein, *B3* acid detergent soluble protein, *C* acid detergent insoluble protein.

In both WS and DS, the feeding trial lasted 23 days and tannin extract supplementation started at morning milking of day 0. To ensure correct feeding, the farmer was the only person aware of the treatment groups allocation. Blinding was used in the next steps of experimental process.

### Feedstuff sampling and analyses

During both experiments, samples of concentrates, hay, and pasture or dry stubble were collected weekly, vacuum-packed and stored at − 20 °C. The weekly subsamples were then pooled in order to get a representative sample for each feed.

Ether extract, CP, and ash were determined according to AOAC^16^ methods 920.39, 976.06, and 942.05, respectively. Protein fractions were calculated according to the Cornell Net Carbohydrate and Protein System, as modified by Licitra et al.^17^. The analyses of NDF, ADF, and ADL were performed following the method of Van Soest et al.^18^. Total phenolic compounds and total tannins were analysed according to the procedure of Makkar et al.^14^, as modified by Luciano et al.^19^. Fatty acid profile of feeds was determined through a one-step extraction-transesterification with chloroform and sulfuric acid (2% in methanol, vol/vol) as methylation reagent^20^. Gas-chromatograph (ThermoQuest) equipment and settings were the same as described by Natalello et al.^21^.

### Milk sampling and analyses

Milk sampling was performed at the following days of trial: − 2, − 1, 1, 2, 3, 4, 5, 8, 11, 15, 18 and 23. Cows were individually milked twice a day (0700 h and 1700 h) with a milking machine (43 kPa vacuum, 60 pulsations/min). The milk of each cow was sampled individually. Each sampling day included the milk of two subsamples (250 mL) from two consecutive milkings: the evening milking and the following morning milking. The evening milking subsample was stored refrigerated until the next morning. To get a representative daily sample, the two subsamples were pooled according to the proportion between the milk amount recorded at the respective evening and morning milking. Analyses of proximate composition, somatic cells count (SCC), colour parameters, laboratory cheese yield (LCY), and milk coagulation properties (MCP) were immediately performed on fresh milk samples. The aliquots for antioxidant capacity assays and FA profile determination were stored at − 80 °C. Before freezing, sodium azide was added to the aliquots for FA profile determination, to a final concentration of 0.3 g/L.

Fat, lactose and protein contents in milk, and milk urea nitrogen (MUN) were analysed with a Milkoscan FT 1 (Foss, Hillerod), according to ISO 9622^22^. On the same aliquot, SCC was determined by using a BacSomatic (Foss), according to ISO 13366-2^23^.

Milk colour parameters were measured using a Minolta CM-2022 portable spectrophotometer (d/8° geometry) in the CIE L*a*b space (illuminant A, 10° standard observer). Measured parameters were lightness (L*), redness (a*), yellowness (b*), chroma (C*), hue angle (H*), and the reflectance spectra between 400 and 700 nm.

The LCY and laboratory dry matter cheese yield (LDMCY) were determined according to the method of Hurtaud et al.^24^, using a commercial liquid calf rennet (105 IMCU/mL, 80% chymosin and 20% pepsin; Biotec Fermenti S.r.l.). The MCP of milk were analysed using a formagraph (Maspres and Foss Italia), following the method of Zannoni and Annibaldi^25^. Determined parameters were clotting time (time needed for the beginning of coagulation), firming time (time needed to reach 20 mm of amplitude on the chart), and curd firmness (i.e., amplitude of the chart in mm) after 30 min and after two times clotting time. A detailed procedure for LCY, LDMCY, and MCP is reported in Menci et al.^12^.

The antioxidant capacity of the hydrophilic fraction of milk was assessed by ferric reducing antioxidant power (FRAP) and Trolox-equivalent antioxidant capacity (TEAC) assays. Milk was pre-treated before analyses, as follows. Defrosted samples were vortexed thoroughly and 100 µL of milk was transferred in a 1.5-mL tube with 900 µL of water and 200 µL of hexane. After centrifugation at 1500×*g* for 10 min at 4 °C, two aliquots of 50 µL and 20 µL of the lower phase were transferred in plastic tubes and analysed in duplicate for FRAP assay and TEAC assay, respectively. The FRAP assay was performed following Benzie and Strain^26^ method, with modifications. A solution 50:5:5:6 of pH 3.6 acetate buffer (300 mM sodium acetate trihydrate in 1.6% acetic acid), 0.01 M TPTZ [2,4,6-tris(2-pyridyl)-s-triazine] in 0.04 M hydrochloric acid, 0.02 M ferric chloride hexahydrate, and distilled water was made, and 1650 µL of this solution was added to samples. After incubation in water bath at 37 °C for 60 min, absorbance at 593 nm was read using a double beam UV/Vis spectrophotometer (UV-1601, Shimadzu Corporation). An external calibration curve was prepared using 1 mM ferrous sulphate heptahydrate, and FRAP values were expressed as mmol of Fe^2+^ equivalent per L of milk. The TEAC assay was performed according to Re et al.^27^, with some modifications. A stable radical solution 1:1 of 14 mM ABTS (2,2-azinobis-3-ethylbenzothiazoline-6-sulfonic acid) and 4.9 mM potassium persulfate was incubated in the dark at room temperature for 12–14 h and then diluted to an absorbance of 0.75 at 734 nm. After adding 2 mL of diluted radical solution, samples were incubated at 30 °C for 60 min and absorbance at 734 nm was read using UV-1601 spectrophotometer. The reduction of absorbance was compared to a blank and an external seven-points calibration curve was prepared using 2.5 mM Trolox solution. Results are expressed as mmol of Trolox equivalent per L of milk.

The FA profile of experimental milk was determined by gas chromatographic analysis of fatty acid methyl esters, after fat separation according to the method B described by Feng et al.^28^, with some modification. Briefly, the top fat-cake layer of 50-mL milk samples was removed after centrifugation at 13,000×*g* for 30 min at 4 °C. Fat was then transferred in a 2-mL tube, let melt at room temperature for 30 min and centrifuged at 19,300×*g* for 20 min. About 50 mg of the top lipid-layer was then transferred in a glass tube for transesterification, following the method described by Christie^29^, with modifications. Briefly, 1 mL of 0.5 N methanolic sodium methoxide was added, and samples were vortexed for 3 min. After a 5 min pause, 2 mL of hexane was added, and samples were vortexed for 30 s. The upper phase was then transferred in a 2-mL vial, a little spoon of sodium sulphate was added, and vials were then stored at − 20 °C. Gas-chromatograph (ThermoQuest) equipment and settings were the same as described by Natalello et al.^21^. Moreover, the separation of C18:1 *t*10 and C18:1 *t*11 was achieved by isothermal analysis at 165 °C.

### Calculations and statistics

The reflectance spectrum at wavelengths between 530 and 450 nm was elaborated as done by Priolo et al.^30^ to calculate the integral value (I_450–530_). Before statistical analysis, SCC data was transformed to log10/mL to obtain normalized distribution.

All data from WS and DS trials were statistically elaborated separately using a mixed model ANOVA for repeated measures of IBM SPSS 21 For Analytics, with individual milk sample as statistical unit, using formula (1).

1 $${y}_{ijkl}=\mu +{T}_{i}+{D}_{j}+{\left(T\times D\right)}_{ij}+{C}_{k}\left({T}_{i}\right)+ {BX}_{ik}+{e}_{ijkl},$$

where *y*_*ijkl*_ is the observation, µ is the overall mean, *T*_*i*_ is the fixed effect of treatment (*i* = 1–2), *D*_*j*_ is the fixed effect of sampling day (*j* = 1–10), (*D* × *T*)_*ij*_ is the interaction between diet and sampling time, *C*_*k*_ is the random effect of the cow nested within the treatment (*k* = 1–7), *BX*_*ik*_ is the covariate adjustment for each cow, and *e*_*ijkl*_ is the residual error. The milk sampled in the two days before the beginning of the trial (i.e., sampling days − 2 and − 1) was analysed and averaged to constitute the covariate for statistical elaboration. In addition, statistical elaboration was adjusted for a covariate composed of DIM. For individual FA, fat content was included as covariate in the statistical model. When the effect of the covariate had *P* ≤ 0.100, it was removed from the statistical model. Multiple comparisons among means were performed using the Tukey’s test and differences between treatment means were considered to be significant at *P* ≤ 0.050 and a trend towards significance at *P* ≤ 0.100. All the results showed in tables refer to estimated marginal means.

## Results

### WS experiment

Table 2 shows the results on proximate composition, physical parameters, and antioxidant capacity of WS milk. Dietary tannins did not affect (*P* > 0.100) milk yield, milk composition, and colour parameters. Likewise, milk cheesemaking parameters and antioxidant capacity did not differ (*P* > 0.100) between the two dietary groups.

**Table 2:** Effect of dietary tannin extract on physicochemical properties of milk in wet season experiment.

Item^a^	Treatment^b^ (T)		SEM	*P* value^c^		
	CON	TAN		T	Day (D)	T × D
Milk yield, kg/day	11.60	13.21	0.616	ns	*	ns
ECM, kg/day	12.84	14.51	0.643	ns	†	ns
Fat, g/100 g	3.908	3.971	0.087	ns	**	ns
Lactose, g/100 g	4.584	4.611	0.029	ns	**	ns
Protein yield, g/day	460	497	18.6	ns	*	ns
Protein, g/100 g	3.927	3.918	0.051	ns	ns	ns
Casein, g/100 g	3.042	3.037	0.047	ns	ns	ns
Urea, mg/dL	33.00	33.40	0.625	ns	***	†
SCC, log_10_/mL	2.843	2.898	0.040	ns	ns	ns
**Colour parameters**						
L*	70.00	70.32	0.295	ns	***	ns
a*	− 1.434	− 1.491	0.056	ns	**	ns
b*	3.59	3.14	0.237	ns	***	ns
C*	3.96	3.65	0.204	ns	***	ns
H*	115.2	120.0	2.24	ns	***	ns
I_450–530_	− 264	− 234	12.0	ns	***	ns
**Cheesemaking properties**						
LCY, g/kg	263.9	265.5	4.42	ns	ns	ns
LDMCY, g/kg	83.33	83.88	0.725	ns	***	ns
R, min:s	24:15	24:23	0:42	ns	***	ns
K20, min:s	5:33	5:10	0:20	ns	ns	ns
A30, mm	30.2	27.6	2.75	ns	*	ns
A2R, mm	44.9	45.2	1.00	ns	ns	ns
**Antioxidant capacity, mmol/L**						
FRAP	4.90	4.80	0.130	ns	***	ns
TEAC	10.89	9.87	0.427	ns	***	ns

^a^L* lightness, a* redness, b* yellowness, C* chroma, H* hue angle, *I*_*450–530*_ integral value of the absorbance spectrum from 450 to 530 nm, *LCY* laboratory cheese yield, *LDMCY* laboratory dry matter cheese yield, *R* clotting time, *K20* firming time, *A30* curd firmness after 30 min, *A2R* curd firmness after two times R, *FRAP* ferric reducing antioxidant power, *TEAC* Trolox-equivalent antioxidant capacity, *ECM* energy corrected milk, *SCC* somatic cells count.

^b^*CON* control group, *TAN* group receiving 150 g/head × day of tannin extract.

^c^*ns*  ≥ 0.100; ^†^< 0.100; *< 0.050; **< 0.010; ***< 0.001.

**Figure 1:**
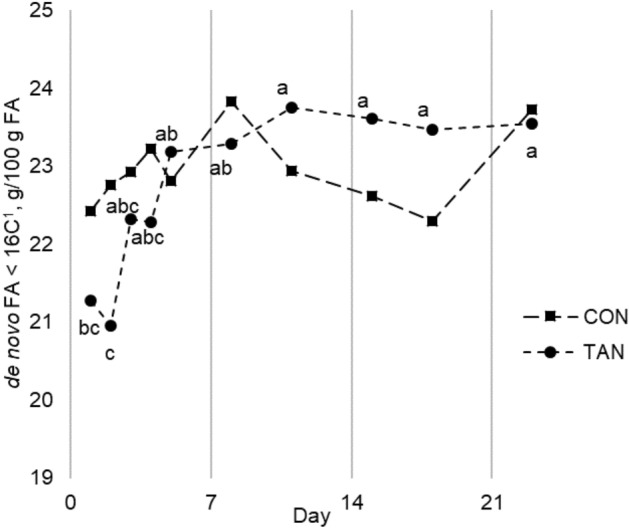
Concentration of de novo fatty acids (FA) in cow milk during wet season experiment. *CON* control group, *TAN* group receiving 150 g/head per day of tannin extract. ^1^De novo FA < 16C = sum of C4:0. C6:0. C8:0. C10:0. C12:0 and C14:0. ^a,b,c^Points with different letters differ at *P* < 0.050, within TAN group. There was no difference between points within CON group.

**Figure 2:**
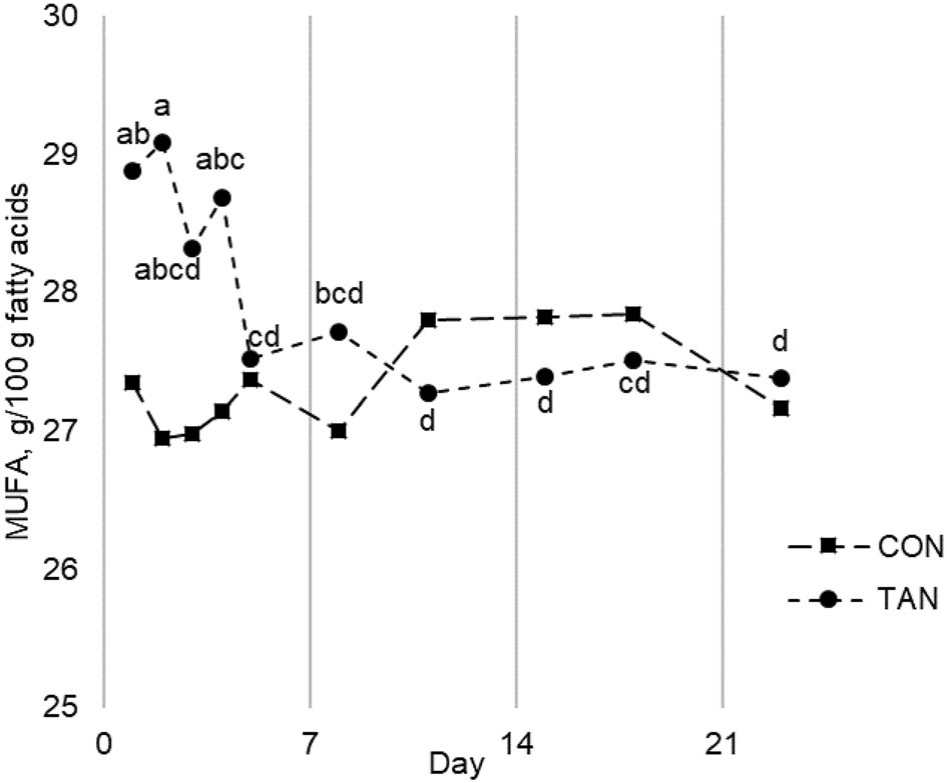
Sum of monounsaturated fatty acids (MUFA) concentrations in cow milk during wet season experiment. *CON* control group, *TAN* group receiving 150 g/head per day of tannin extract. ^a,b,c,d^Points with different letters differ at *P* < 0.050, within TAN group. There was no difference between points within CON group.

Concerning FA concentration (Table 3), no differences were found between dietary groups for almost all FA, but we observed a different kinetic of some FA along the trial. Tannin supplementation depressed (*P* = 0.021) the concentration of de novo FA with less than 16 C, particularly C8:0 (*P* = 0.033), C10:0 (*P* = 0.010), C12:0 (*P* = 0.030) and C14:0 (*P* = 0.082), but only in the first two days of treatment (Fig. 1). Moreover, a higher proportion of C18:1 *c*9 (*P* = 0.047), C18:1 *t*9 (*P* = 0.001), C18:1 *c*11 (*P* = 0.040) was observed in TAN milk in the first days of tannin administration, reflecting on total monounsaturated fatty acids (MUFA) concentration (*P* = 0.028; Fig. 2). On the other hand, FA profile of CON milk did not vary statistically throughout the observation period.

**Table 3:** Effect of dietary tannin extract on fatty acid profile of milk in wet season experiment.

Item^a^	Treatment^b^ (T)		SEM	*P* value^c^		
	CON	TAN		T	Day (D)	T × D
C4:0	1.871	1.860	0.038	ns	ns	ns
C6:0	1.643	1.626	0.031	ns	ns	ns
C8:0	1.257	1.238	0.027	ns	*	*
C10:0	3.218	3.127	0.078	ns	***	**
C11:0	0.370	0.380	0.010	ns	***	ns
C12:0	3.943	3.823	0.098	ns	***	*
C13:0	0.255	0.250	0.008	ns	***	ns
C14:0	10.99	11.06	0.137	ns	***	†
C14:0 *iso*	0.159	0.154	0.005	ns	***	ns
C14:1 *c*9	0.843	0.907	0.024	ns	***	ns
C15:0	1.324	1.304	0.020	ns	***	ns
C15:0 *iso*	0.308	0.309	0.005	ns	***	ns
C15:0 *anteiso*	0.648	0.638	0.014	ns	***	ns
C16:0	24.83	24.54	0.271	ns	ns	ns
C16:0 *iso*	0.325	0.320	0.008	ns	**	ns
C16:1 *t*9	0.157	0.158	0.007	ns	**	ns
C16:1 *c*9	1.223	1.214	0.024	ns	***	ns
C17:0	0.577	0.560	0.010	ns	**	ns
C17:0 *iso*	0.412	0.419	0.009	ns	**	ns
C17:0 *anteiso*	0.605	0.575	0.015	ns	***	ns
C18:0	10.18	9.73	0.155	ns	***	ns
C18:1 *t*6 + *t*7 + *t*8	0.186	0.188	0.005	ns	*	ns
C18:1 *t*9	0.277	0.272	0.005	ns	***	***
C18:1 *t*10	0.266	0.271	0.014	ns	ns	ns
C18:1 *t*11	2.870	2.980	0.082	ns	ns	ns
C18:1 *c*6	0.722	0.729	0.024	ns	†	ns
C18:1 *c*9	19.86	20.24	0.329	ns	†	*
C18:1 *c*11	0.523	0.527	0.012	ns	***	*
C18:1 *c*12	0.202	0.193	0.005	ns	**	ns
C18:1 *c*13	0.061	0.059	0.002	ns	ns	ns
C18:1 *c*14	0.357	0.362	0.011	ns	†	ns
C18:2 *t*8*c*13	0.268	0.260	0.010	ns	***	ns
C18:2 *t*9*c*13	0.501	0.498	0.016	ns	**	ns
C18:2 *c*9*c*12 (LA)	2.306	2.389	0.061	ns	***	ns
C18:2 *c*9*t*11 (RA)	1.444	1.523	0.047	ns	**	ns
C18:3 *c*9*c*12*c*15	1.114	1.134	0.025	ns	***	ns
C20:0	0.173	0.161	0.004	ns	***	ns
C20:3 *n-*6	0.099	0.093	0.004	ns	*	ns
C20:4 *n-*6	0.152	0.155	0.005	ns	***	ns
C20:5 *n-*3	0.062	0.057	0.001	ns	***	ns
C22:0	0.129	0.122	0.003	ns	***	ns
C22:5 *n-*3	0.138	0.129	0.005	ns	*	ns
C23:0	0.045	0.040	0.002	ns	**	ns
C24:0	0.091	0.087	0.003	ns	***	ns
Σ de novo FA < 16C	22.97	22.70	0.368	ns	***	*
Σ SFA	58.56	57.10	0.554	ns	ns	ns
Σ MUFA	27.27	28.04	0.397	ns	ns	*
Σ PUFA	6.24	6.43	0.119	ns	†	ns
Σ OCFA	2.937	2.922	0.042	ns	***	ns
Σ BCFA	2.469	2.405	0.053	ns	***	ns
Σ *iso*-FA	1.214	1.192	0.026	ns	***	ns
Σ *anteiso*-FA	1.255	1.211	0.028	ns	***	ns
SFA/PUFA	9.56	9.05	0.245	ns	†	ns
*n*-6/*n*-3 PUFA	2.017	2.073	0.046	ns	***	ns
C18:1 *t*10/*t*11	0.100	0.097	0.007	ns	ns	ns
BHI	7.76	7.96	0.194	ns	*	ns
RA / LA	0.649	0.660	0.022	ns	***	ns
DSI C14	0.072	0.076	0.002	ns	***	ns

^a^*De novo FA < 16C* sum of C4:0, C6:0, C8:0, C10:0, C12:0 and C14:0, *SFA* saturated fatty acids, *MUFA* monounsaturated fatty acids, *PUFA* polyunsaturated fatty acids, *OCFA* odd-chain fatty acids, *BCFA* branched-chain fatty acids, *BHI* biohydrogenation intermediates, *DSI C14* desaturation index, calculated as C14:1 *c*9/(C14:0 + C14:1 *c*9).

^b^*CON* control group, *TAN* group receiving 150 g/head × day of tannin extract.

^c^*ns *≥ 0.100; ^†^< 0.100; *< 0.050; **< 0.010; ***< 0.001.

The sampling day effect was significant (*P* < 0.050) for almost all the parameters analysed.

### DS experiment

Proximate composition, physical parameters, antioxidant capacity, and cheesemaking properties of milk in the DS experiment are shown in Table 4. Milk from TAN group had 10% lower (*P* = 0.042) urea nitrogen compared to CON group. Tannin extract supplementation tendentially increased FRAP (*P* = 0.056) and TEAC (*P* = 0.083) values in milk.

**Table 4:** Effect of dietary tannin extract on physicochemical properties of milk in dry season experiment.

Item^a^	Treatment^b^ (T)		SEM	*P* value^c^		
	CON	TAN		T	Day (D)	T × D
Milk yield, kg/day	12.58	12.99	0.115	ns	***	ns
ECM, kg/day	12.98	13.51	0.173	ns	***	ns
Fat, g/100 g	3.81	3.99	0.113	ns	ns	ns
Lactose, g/100 g	4.669	4.630	0.024	ns	ns	ns
Protein yield, g/day	409.8	416.4	3.03	ns	*	ns
Protein, g/100 g	3.386	3.353	0.016	ns	†	ns
Casein, g/100 g	2.569	2.533	0.014	ns	*	ns
Urea, mg/dL	29.24	26.53	0.533	*	**	ns
SCC, log_10_/mL	2.963	3.014	0.038	ns	***	†
**Colour parameters**						
L*	67.32	68.04	0.547	ns	**	ns
a*	− 1.980	− 1.907	0.062	ns	***	ns
b*	0.32	0.64	0.171	ns	***	ns
C*	2.51	2.59	0.100	ns	*	ns
H*	165.4	159.2	3.64	ns	**	ns
I_450–530_	− 73.1	− 86.9	7.26	ns	***	ns
**Cheesemaking properties**						
LCY, g/kg	240.0	245.0	2.86	ns	***	ns
LDMCY, g/kg	73.63	73.96	0.580	ns	ns	ns
R, min:s	19:38	19:51	0:27	ns	***	ns
K20, min:s	5:04	5:29	0:16	ns	*	ns
A30, mm	35.4	30.8	1.64	ns	***	ns
A2R, mm	39.3	39.2	1.26	ns	ns	ns
**Antioxidant capacity, mmol/L**						
FRAP	3.31	3.94	0.122	†	***	ns
TEAC	8.82	10.21	0.365	†	***	ns

^a^L* lightness, a* redness, b* yellowness, C* chroma, H* hue angle, *I*_*450–530*_ integral value of the absorbance spectrum from 450 to 530 nm, *LCY* laboratory cheese yield, *LDMCY* laboratory dry matter cheese yield, *R* clotting time, *K20* firming time, *A30* curd firmness after 30 min, *A2R* curd firmness after two times R, *FRAP* ferric reducing antioxidant power, *TEAC* Trolox-equivalent antioxidant capacity, *ECM* energy corrected milk, *SCC* somatic cells count.

^b^*CON* control group, *TAN* group receiving 150 g/head × day of tannin extract.

^c^ns ≥ 0.100; ^†^< 0.100; *< 0.050; **< 0.010; ***< 0.001.

The FA profile of DS milk is reported in Table 5. Dietary tannins decreased (*P* < 0.001) branched-chain fatty acids (BCFA), particularly C15:0 *anteiso*, C16:0 *iso*, and C17:0 *iso* (*P* = 0.009, *P* = 0.007, and *P* = 0.040, respectively). Milk from TAN group had tendentially lower (*P* = 0.063) C18:1 *t*10 concentration, resulting in tendentially lower (*P* = 0.093) C18:1 *t*10 to C18:1 *t*11 ratio (*t*10/*t*11). Also, C18:2 *c*9*t*11 concentration was lower (*P* = 0.043) and C18:2 *c*9*c*12 concentration tended to be higher (*P* = 0.057) in TAN milk. As a result, C18:2 *c*9*t*11 to C18:2 *c*9*c*12 ratio (RA/LA) was lower (*P* = 0.044) in milk from cows given tannin extract.

**Table 5:** Effect of dietary tannin extract on fatty acid profile of milk in dry season experiment.

Item^a^	Treatment^b^ (T)		SEM	*P* value^c^		
	CON	TAN		T	Day (D)	T × D
C4:0	1.812	1.805	0.043	ns	†	†
C6:0	1.529	1.454	0.025	ns	***	ns
C8:0	0.970	0.932	0.019	ns	*	†
C10:0	2.099	2.093	0.048	ns	†	ns
C11:0	0.256	0.238	0.006	ns	ns	†
C12:0	2.468	2.475	0.054	ns	†	ns
C13:0	0.154	0.148	0.003	ns	ns	ns
C14:0	9.40	9.28	0.125	ns	†	ns
C14:0 *iso*	0.238	0.222	0.007	ns	*	ns
C14:1 *c*9	0.794	0.727	0.015	ns	***	**
C15:0	1.251	1.228	0.018	ns	**	ns
C15:0 *iso*	0.420	0.399	0.007	ns	**	ns
C15:0 *anteiso*	0.792	0.725	0.010	**	***	ns
C16:0	26.15	26.30	0.147	ns	ns	ns
C16:0 *iso*	0.495	0.441	0.008	**	*	ns
C16:1 *t*9	0.080	0.078	0.003	ns	***	ns
C16:1 *c*9	1.437	1.483	0.022	ns	***	ns
C17:0	0.731	0.735	0.009	ns	***	ns
C17:0 *iso*	0.488	0.457	0.006	*	***	ns
C17:0 *anteiso*	0.735	0.734	0.004	ns	***	ns
C18:0	10.45	10.54	0.137	ns	**	ns
C18:1 *t*6 + *t*7 + *t*8	0.179	0.175	0.005	ns	*	ns
C18:1 *t*9	0.327	0.315	0.004	ns	†	ns
C18:1 *t*10	0.270	0.240	0.007	†	***	ns
C18:1 *t*11	1.653	1.603	0.020	ns	***	†
C18:1 *c*6	0.493	0.459	0.011	ns	***	ns
C18:1 *c*9	25.32	25.20	0.312	ns	*	ns
C18:1 *c*11	0.622	0.649	0.008	ns	*	*
C18:1 *c*12	0.249	0.242	0.008	ns	**	†
C18:1 *c*13	0.050	0.060	0.003	ns	*	ns
C18:1 *c*14	0.230	0.221	0.005	ns	***	ns
C18:2 *t*8*c*13	0.169	0.153	0.004	†	***	ns
C18:2 *t*9*c*13	0.106	0.108	0.007	ns	*	ns
C18:2 *c*9*c*12 (LA)	2.632	2.699	0.058	†	*	ns
C18:2 *c*9*t*11 (RA)	0.851	0.791	0.011	*	***	ns
C18:3 *c*9*c*12*c*15	0.360	0.381	0.015	ns	***	ns
C20:0	0.241	0.248	0.003	ns	**	†
C20:3 *n-*6	0.100	0.104	0.002	ns	***	ns
C20:4 *n-*6	0.140	0.140	0.003	ns	***	ns
C20:5 *n-*3	0.075	0.082	0.002	ns	**	ns
C22:0	0.138	0.148	0.004	ns	***	ns
C22:5 *n-*3	0.102	0.105	0.003	ns	***	ns
C23:0	0.064	0.064	0.002	ns	**	ns
C24:0	0.041	0.059	0.003	†	***	ns
Σ de novo FA < 16C	18.28	18.06	0.273	ns	†	ns
Σ SFA	55.13	55.52	0.336	ns	**	ns
Σ MUFA	32.31	31.83	0.321	ns	*	ns
Σ PUFA	4.720	4.790	0.081	ns	*	†
Σ OCFA	2.515	2.440	0.035	ns	***	ns
Σ BCFA	3.185	2.960	0.025	***	ns	ns
Σ *iso*-FA	1.600	1.500	0.016	**	ns	ns
Σ *anteiso*-FA	1.532	1.454	0.011	**	**	ns
SFA/PUFA	11.89	11.80	0.260	ns	*	ns
*n*-6/*n*-3 PUFA	5.75	5.55	0.101	ns	***	*
C18:1 *t*10/*t*11	0.172	0.156	0.004	†	***	ns
BHI	5.297	5.175	0.059	ns	***	ns
RA/LA	0.329	0.301	0.006	*	***	ns
DSI C14	0.075	0.075	0.001	ns	*	ns

^a^*De novo FA < 16C* sum of C4:0, C6:0, C8:0, C10:0, C12:0 and C14:0, *SFA* saturated fatty acids, *MUFA* monounsaturated fatty acids, *PUFA* polyunsaturated fatty acids, *OCFA* odd-chain fatty acids, *BCFA* branched-chain fatty acids, *BHI* biohydrogenation intermediates, *DSI C14* desaturation index, calculated as C14:1 *c*9/(C14:0 + C14:1 *c*9).

^b^*CON* control group, *TAN* group receiving 150 g/head × day of tannin extract.

^c^ns ≥ 0.100; ^†^< 0.100; *< 0.050; **< 0.010; ***< 0.001.

A significant interaction of dietary treatment with sampling day was found for few FA (*P* < 0.050), reflecting a different evolution of the concentration throughout the trial in the two groups. The concentration of C14:1 *c*9 in CON milk was higher at the 23rd day of trial than in the first four days, whereas in TAN milk there were no changes along time (*P* = 0.004). The concentration of C18:1 *c*11 in TAN milk after two weeks of treatment (i.e., at sampling day 15, 18 and 23) was higher than it was at the first sampling day, whereas no significative variation was observed in CON milk (*P* = 0.021). Finally, *n*-6 PUFA to *n*-3 PUFA ratio (*n*-6/*n*-3) increased, peaked at the 11th day and then decreased in both the experimental groups, but in CON milk the highest value differed only from the first five sampling days whereas in TAN milk the highest value differed from all but the fifth’s day observations (*P* = 0.033). Anyway, neither the concentrations of C14:1 *c*9 and C18:1 *c*11 nor *n*-6/*n*-3 statistically differed between CON and TAN milk on any of the sampling day.

The sampling day effect was significant (*P* < 0.050) for almost all the parameters analysed.

## Discussion

The significance of sampling day effect found in this study was due to environmental factors falling outside our experimental design, so it will not be discussed further. Probably, the periodical monitoring of milk throughout the 23 days of trial, combined with the continuous free ranging of cows, made our experimental design sensitive to weather variations. For example, in July, during DS experiment, the temperature leap led to a range of average temperature between 19.5 and 32 °C.

### Milk proximate composition

In the present study, the effect of dietary tannin supplementation on milk yield and its main constituents was not significant, regardless of the season. In the last decade, most scientific articles reported no improvement in milk yield, or fat and protein contents from cows eating different sources of tannin^31–33^. Henke et al.^34^ reported an increase in milk fat content in cows supplemented for 21 days with 3% DMI of quebracho tannin extract, but the effect was combined with a lower milk yield. Even, increasing doses of tannin have been reported to have a negative linear effect on cows' milk protein content^35,36^.

In addition, our results suggest that the lack of effectiveness of dietary tannins is constant from the 1st to the 23rd day of administration in dairy cows. Consistently, Denninger et al.^37^ did not observe any variations in cow milk yield, and fat and protein contents throughout 3 days of dietary *Acacia mearnsii* (De Wild) tannin extract supplementation (14.7 g of total tannins per kg of DM).

On the other hand, a reduction in MUN of TAN group was expected, as it is reported in several studies on dairy cows involving different tannin sources, such as quebracho^34^, bayberry, *Acacia mangium* Willd. and valonia^32^ or sainfoin (*Onobrychis viciifolia* Scop.)^38^. The reduction in MUN is potentially positive for the environment, as it is an indicator of urinary N excretion^39^. In extensive farming systems, this effect is desirable right in green season, as the high degradable protein content of herbage maximizes the N emission^5^. The lack of an effect on MUN in WS experiment may be due to the relatively low dose of tannin supplementation, as in the studies where a significant effect was reported cows ingested about 3% DMI of tannin^32,34,38^. Also, the plant species from which tannins are extracted are a well-known limit to studies comparison^40^. Aguerre et al.^41^ fed cows a tannin extract very similar to ours at increasing doses and no evident MUN reduction occurred at supplementations lower than 1.8% DMI. Anyway, a high intake of tannins could have detrimental consequences on animal performance^35^ and could be economically unpractical on commercial farm.

On the contrary, in DS experiment we observed a constant reduction in MUN. Probably, the dose of tannin extract we supplemented in cow’s diet was not enough to modulate ruminal N metabolism when CP intake is as high as it was in WS experiment. Aguerre et al.^35^ did not found any interaction between dietary treatment and CP intake on cow’s N partitioning when administrating 0.45% up to 1.8% DMI of quebracho-chestnut tannin mixture. However, the two dietary CP levels they compared were 15.3% and 16.6% DM, whereas in our study the estimated CP levels were 13.9% and 19.9% DM in DS and WS experiment, respectively.

### Milk cheesemaking aptitude

In WS experiment, the lack of an effect on milk cheesemaking aptitude was not surprising, considering that fat, protein and casein contents, and even MUN were not affected by dietary tannin extract supplementation. This occurred also in DS experiment, where the reduction in MUN could have been linked with other parameters related to cheesemaking properties^42^. In accordance with our findings, Herremans et al.^43^ concluded in a meta-analysis study that dietary tannins do not have any effect on N use efficiency in dairy cattle, except for the reduction of urea emissions.

In a previous study from the same experiment, investigating the effect of dietary tannin extract on cheese quality^12^, we found no effects on cheesemaking aptitude after 23 days of dietary trial. With the findings of the present study, we can add that this lack of effect is consistent from the 1st to the 23rd day of administration.

Kalber et al.^44^ found that milk from cows eating buckwheat (*Fagopyrum esculentum* Moench) silage had a shorter clotting time compared to milk from cows eating chicory (*Cichorium intybus* L.) or ryegrass (*Lolium multiflorum* Lam.) silages. The three treatments significantly differed for condensed tannins intake, with 6.1 g/day for cows eating buckwheat and about 2.2 g/day for cows eating chicory or ryegrass, but these intake levels seem too low to confidently attribute them the observed effect. At the best of our knowledge, no other study is available for comparison, and we cannot speculate if dietary tannins at doses higher than 1% DMI could exert an effect on cow milk cheesemaking properties. Also, the few scientific articles investigating dietary tannins effect on clotting time of ewe milk are discordant, reporting no effect^45^ or even longer clotting and firming times^46^ with ewes eating 1.6% DMI of tannin extracts. Anyway, literature suggests that a plant specific effect may occur, and results may vary administrating different tannin sources.

### Antioxidant capacity of milk hydrophilic fraction

In our study, we investigated both the reducing power and the radical scavenging capacity of skimmed milk. Avila et al.^36^ did not observe an improvement in milk reducing power when cows’ diet was supplemented with 5 up to 20 g/kg DM of *A. mearnsii* tannin extract. Unlike them, we analysed not-deproteinized milk, to also include the antioxidant activity of caseins and whey proteins^47^. Interestingly, although we observed no variation in protein and casein contents of milk, the dietary tannin extract supplementation tended to increase both the reducing power and the radical scavenging capacity of defatted milk in DS experiment. Although the antioxidant activity of polyphenolic compounds is well-known^48^, is not yet clear how they could improve the antioxidant status of animal products. Probably, tannins had an indirect effect preserving other antioxidants (e.g., vitamin E, vitamin C) during digestion, or low molecular-weight molecules derived from tannins digestion could have been absorbed in the intestine and therefore exerted their antioxidant activity in milk^49^. The lack of effect in WS experiment could be explained by the already relatively high TEAC and FRAP values. Probably, the diet of cows in WS experiment had an antioxidants content high enough to suffice for milk oxidative stability, without the contribution of supplementary tannins bioactivity. Indeed, grazing pasture is commonly reported to increase the content of antioxidants in milk^3^, and b* and I_450–530_ values we found in WS milk indicated a higher content of carotenoids^50^, compared to DS milk.

### Milk fatty acid profile

An effect on microbial and rumen preformed FA concentrations in milk is generally expected with dietary tannins, because of their well-known activity against rumen BH^51^. This is in contrast with the results of WS experiment, were neither odd- and branched-chain FA nor the C18:1 and C18:2 isomers differed between CON and TAN milk. Probably, as suggested above, the tannin extract supplementation dose used in the present study was not enough to exert an effect on milk quality during the WS experiment.

Interestingly, the effect of dietary tannins on some MUFA and de novo FA concentrations right after the beginning of administration was never observed before. Recently, Denninger et al.^37^ investigated the effect of *A. mearnsii* tannin extract supplementation in the first 3 days of administration. They observed a decrease in microbial-derived FA in milk starting from the second day of trial, indicating a quite rapid effectiveness of dietary tannins against microbial rumen activity. Likewise, in WS experiment we observed an immediate response of milk FA profile to dietary tannins administration, even if concerning different FA compared to Denninger et al.^37^. As C18:1 *c*9 can undergo BH in the rumen^52^ and dietary supplementation of C18:1 *c*9 is reported to reduce mammary FA synthesis^53^, we hypothesized th at, in WS experiment, dietary tannins impaired the ruminal metabolism of C18:1 *c*9, with consequent increase in C18:1 *c*9 intestinal flow and reduction in de novo FA synthesis. Milk C18:1 *c*9 also results from the activity of mammary Δ9-desaturase, but we found no variation of desaturation index throughout the WS experiment. However, this effect against rumen BH vanished soon, probably indicating a rapid adaptation of ruminal microbiota to dietary tannins, as already suggested by Toral et al.^54^ observing ewe milk.

The different conditions of DS experiment modified the effect of dietary tannin extract on milk FA profile, compared to WS experiment. As recently reviewed by Frutos et al.^51^, a decrease of bacterial FA concentration, such as odd-chain fatty acids (OCFA) and BCFA, is often reported in studies investigating dietary tannins effect on rumen digesta. Interestingly, in DS experiment we observed a decrease in both main *iso-* and *anteiso-*FA in TAN milk, whereas milk’s OCFA did not varied between dietary treatments. Likewise, Denninger et al.^37^ reported a decrease in some BCFA in cow milk after *A. mearnsii* tannin extract feeding and no effect on milk’s OCFA. As changes of bacterial FA proportions in milk likely reflect shifts in rumen microbial community, and the bacterial FA synthesis is species-specific^55^, the effect observed could be explained by the different sensitivity of rumen microorganisms to tannins. Probably, in DS experiment, the ruminal microorganisms enriched in BCFA were more sensitive to tannins bioactivity than those enriched in OCFA. Indeed, cellulolytic and amylolytic bacteria are reported to be enriched in BCFA and OCFA, respectively^55^, and Díaz Carrasco et al.^56^ documented the ability of tannins to modify the cellulolytic:amylolytic bacteria balance in the rumen.

In DS experiment, the effect of dietary tannin extract on *t*10/*t*11 and RA/LA seems to indicate an impaired rumen BH. Indeed, C18:1 *t*10, C18:1 *t*11 and C18:2 *c*9*t*11 are not dietary FA and they form in the rumen by microorganism activity^57^. The reduced RA/LA may indicate a slowdown in the first steps of BH, where C18:2 *c*9*c*12 is isomerized to C18:2 *c*9*t*11^58^. A second source of milk C18:2 *c*9*t*11 is the mammary Δ9-desaturation of C18:1 *t*11^59^. However, an effect of dietary tannin extract on mammary Δ9-desaturase may be ruled out, as desaturation index did not differ between CON and TAN milk in DS experiment. Unfortunately, the extent of the modifications induced by dietary tannins on FA profile in our experiments is hardly relevant in terms of product healthiness.

Our study suggests a different effect of dietary tannins on cows' milk FA profile depending on the grazing season in the Mediterranean. This phenomenon is likely related to the different diet in WS and DS experiment, concerning the green herbage availability, the CP level, the forage to concentrate ratio, the different amount and composition of biohydrogenation precursors, or a combination of all these aspects. Similarly, Menci et al.^8^ found two different tannin extracts (quebracho vs chestnut + quebracho) to reduce *iso*-FA concentration and RA/LA in rumen digesta fermenting a hay substrate, whereas none of these effects was observed fermenting the corresponding green herbage. Different diets are reported to select different microbiota populations in the rumen^60^ and the specific microorganisms can show a different sensitivity to tannins bioactivity^56^. Therefore, the microbiota selected by the diet of DS experiment could have been more sensitive to dietary tannin supplementation. A second hypothesis is that the highly nutritious diet in WS experiment made the rumen microbiota resilient, whereas in DS experiment the microbiota could not adapt to tannin extract supplementation. Indeed, the variations observed in FA profile of DS milk were consistent throughout the whole trial. Anyway, as different rumen microorganisms show a different sensitivity to different kinds of tannin^61^, results may change by varying the source of tannin.

## Conclusions

The dietary supplementation of tannin extract at the dose of 150 g/day in dairy cows showed different effects on milk quality according to the season under Mediterranean climate. No effect on milk quality was observed in WS, whereas in DS the milk from cows eating tannins showed lower BCFA concentration, C18:1 *t*10 to C18:1 *t*11 ratio, and rumenic to linoleic acid ratio. Also, tannin extract supplementation in DS reduced MUN and slight improved the antioxidant capacity of milk. Thus, tannin supplementation in grazing dairy cows was more effective during the dry season, when diet is low in CP and rich in ADF and ADL. Probably, the integration of tannin in the diet should be adapted to the CP or fibre intakes, or both, if the purpose is modifying milk quality. This could have practical implications for a more conscious use of tannin-rich extracts and also other tannin sources such as agro-industrial by-products and forages (especially those from dry areas). Further studies are needed to investigate the effects of longer supplementations or different doses and tannin sources. Finally, a deeper knowledge of the sensitivity of rumen microbiota to tannins could help to explain the variability in dietary tannins effectiveness.

## Data Availability

The datasets used and/or analysed during the current study are available from the corresponding author on reasonable request.

## Abbreviations

ADF: Acid detergent fibre
ADL: Acid detergent lignin
BCFA: Branched-chain fatty acids
BCS: Body condition score
BH: Biohydrogenation
CON: Control group
CP: Crude protein
DIM: Days in milk
DMI: Dry matter intake
DS: Dry season
ECM: Energy corrected milk
FA: Fatty acids
FRAP: Ferric reducing antioxidant power
I_450–530_: Integral value of the absorbance spectrum between 450 and 530 nm
IMCU: International milk clotting units
LCY: Laboratory cheese yield
LDMCY: Laboratory dry matter cheese yield
MCP: Milk coagulation properties
MUFA: Monounsaturated fatty acids
MUN: Milk urea nitrogen
*n*-6/*n*-3: *n-*6 PUFA to *n*-3 PUFA ratio
NDF: Neutral detergent fibre
OCFA: Odd-chain fatty acids
PUFA: Polyunsaturated fatty acids
RA/LA: C18:2 *c*9*t*11 to C18:2 *c*9*c*12 ratio
SCC: Somatic cells count
*t*10/*t*11: C18:1 *t*10 to C18:1 *t*11 ratio
TAN: Group receiving 150 g/head per day of tannin extract
WS: Wet season
